# Generationswandel in der urologischen Versorgungsrealität: Bestandsaufnahme der Deutschen Urologie

**DOI:** 10.1007/s00120-026-02879-4

**Published:** 2026-06-29

**Authors:** Sophie Knipper, Maria-Noemi Welte, Raisa Abrams-Pompe, Laura Bellut, Eva Maria Greiser, Annika Herlemann-Tilke, Karina Müller, Sandra Schönburg, Carolin Siech, Sarah Weinberger, Laura Wiemer, Margarete Teresa Walach

**Affiliations:** 1 Klinik für Urologie, Vivantes Klinikum am Urban, Berlin, Deutschland; 2 Martini-Klinik Prostatakarzinomzentrum, Universitätsklinikum Hamburg-Eppendorf, Hamburg, Deutschland; 3 Klinik für Urologie, Universitätsklinikum, Goethe Universität Frankfurt, Frankfurt am Main, Deutschland; 4 Klinikum Herford, Universitätsklinik für Urologie, Ruhr-Universität Bochum, Campus OWL, Herford, Deutschland; 5 Klinik für Urologie und Kinderurologie, Universitätsklinikum Erlangen, Erlangen, Deutschland; 6 Mathias-Spital, Klinikum Rheine, Rheine, Deutschland; 7 Urologische Klinik und Poliklinik, Campus Großhadern, LMU München, München, Deutschland; 8 Urologische Klinik, Klinikum Bremen Mitte, Bremen, Deutschland; 9 Neuro-Urologie, BG-Klinikum Bergmannstrost, Halle (Saale), Deutschland; 10 Medizinische Fakultät, Martin-Luther-Universität Halle-Wittenberg, Halle (Saale), Deutschland; 11 Urologische Klinik, Charité – Universitätsmedizin Berlin, Berlin, Deutschland; 12 Klinik für Urologie und Urochirurgie, Universitätsmedizin Mannheim, Mannheim, Deutschland

**Keywords:** Bestandsaufnahme, Berufszufriedenheit, Arbeitszeitmodell, Arbeitsbelastung, Generationswandel, Nachwuchssicherung, Status quo analysis, Job satisfaction, Working time model, Workload, Generational shift, Ensuring future workforce

## Abstract

**Hintergrund:**

Die urologische Versorgung in Deutschland steht vor Herausforderungen durch hohe Arbeitsbelastung, dem Wunsch nach flexibleren Arbeitszeiten und der Sicherung des Nachwuchses. Vor diesem Hintergrund wurden Arbeitsbelastung, Arbeitszeitmodelle und Berufszufriedenheit von Urologinnen und Urologen in Deutschland untersucht.

**Methoden:**

Eine bundesweite Online-Umfrage (DGU-/BvDU-/GeSRU-Verteiler) von 10–12/2024 erhob Daten zu Demografie, Qualifikationen, Arbeitsumfeld, -belastung, -zufriedenheit und familiärer Situation unter urologisch tätigen Ärztinnen und Ärzten.

**Ergebnisse:**

Von den 999 Teilnehmenden (66 % männlich, 34 % weiblich, 0,1 % divers) hatten 61 % einen Doktortitel, 8 % waren habilitiert (4 % mit Professorentitel) und 19 % gingen einer wissenschaftlichen Tätigkeit nach. Die Mehrheit (73 %) war operativ tätig, 67 % mit allgemeinurologischem und 36 % mit konservativ-onkologischem Schwerpunkt. 74 % arbeiteten in Vollzeit (≥ 40 h/Woche), 54 % sogar ≥ 50 h/Woche inklusive Überstunden und Bereitschaftsdiensten. Ambulant arbeiteten 53 %, stationär 43 % und 2 % in Wissenschaft, Privatwirtschaft, Industrie oder einer Behörde. 50 % lebten mit mind. einem Kind < 18 Jahren im Haushalt und 31 % hatten Elternzeit genommen. Insgesamt beschrieben 80 % eine hohe/sehr hohe Arbeitsbelastung und 54 % gaben an, ihre tatsächliche Arbeitszeit reduzieren zu wollen. Dennoch waren 56 % zufrieden/sehr zufrieden mit ihrer aktuellen beruflichen Situation.

**Schlussfolgerungen:**

Die Befragung zeigt eine hohe Arbeitsbelastung, bei gleichzeitig großer Zufriedenheit mit dem urologischen Beruf. Der mehrheitliche Wunsch nach Reduktion der tatsächlichen Arbeitszeit zeigt jedoch den Handlungsbedarf für strukturelle Anpassungen auf, damit die Attraktivität der Urologie langfristig gesichert werden kann.

## Abstract (en)

**Background/Objectives:**

Urological care in Germany is facing challenges due to high workload, an increasing desire for more flexible working time models, and the need to secure the next generation of professionals. Against this backdrop, workload, working time models, and job satisfaction among urologists in Germany were examined.

**Materials and methods:**

A nationwide online survey (mailing lists of *Deutsche Gesellschaft für Urologie* [DGU], *Bundesverband der deutschen Urologie* [BvDU], German Society of Residents in Urology [GeSRU]) conducted from October–December 2024 collected data on demographics, qualifications, work environment, workload, job satisfaction, and family situation among practicing urologists.

**Results:**

Of the 999 participants (66% male, 34% female, 0.1% diverse), 61% held a doctoral degree, 8% were habilitated (4% with a professorship), and 19% were involved in research. Most (73%) were surgically active; 67% focused on general urology and 36% on conservative-oncological care. While 74% worked full-time (≥ 40 h/week), 54% reported ≥ 50 h/week including overtime and on-call duties. The majority worked in outpatient settings (53%), 43% in inpatient care, and 2% in research, industry, or public service. Half lived with at least one child under 18. Parental leave had been taken by 31%, with 56% of them for more than 6 months. Overall, 80% reported a high or very high workload, 54% wished to reduce working hours, yet 56% were satisfied or very satisfied with their current job situation.

**Conclusion:**

The survey highlights a high workload combined with still considerable satisfaction with the profession among urologists in Germany. However, the majority desire a reduction in actual working hours, which indicates a need for structural adjustments to ensure the long-term attractiveness of the field of urology.

## Einleitung

Das deutsche Gesundheitssystem steht derzeit vor Herausforderungen, die sich auch in der Urologie in den zentralen Dimensionen Arbeitsbelastung, Arbeitszeitgestaltung und Berufszufriedenheit manifestieren. Strukturelle Veränderungen im Gesundheitswesen, demografischer Wandel und steigende Ansprüche an die Work-Life-Balance wirken sich direkt auf Attraktivität, Nachwuchsgewinnung und Versorgungsqualität aus [1–3].

Ein zentrales Problem stellt die erhebliche Arbeitsbelastung des medizinischen Personals dar, die maßgeblich durch übermäßige administrative Tätigkeiten, unzureichende digitale Infrastruktur sowie strukturelle Personalengpässe verstärkt wird [2]. Diese Faktoren führen zur physischen und psychischen Erschöpfung des Personals und belasten die Funktionsfähigkeit des Gesundheitssystems [1, 3]. Operative Fachrichtungen wie die Urologie sind durch hohe operative Belastung, Notfallbereitschaften, komplexe onkologische Behandlungen und eine zunehmende Spezialisierung zusätzlich betroffen.

Die Auswirkungen dieser Überlastung spiegeln sich in der Berufszufriedenheit wider, die sowohl die Identifikation mit der Fachrichtung als auch die Bewertung der Arbeitsbedingungen umfasst. Angesichts von Reformprozessen, Personalknappheit und einem steigenden Anteil von Frauen im ärztlichen Berufsfeld gewinnt die Förderung der Berufszufriedenheit besondere Relevanz [4]. Dies gilt insbesondere vor dem Hintergrund, dass der ärztliche Beruf traditionell als Berufung verstanden wird, die nicht selten über die Einhaltung regulärer Arbeitszeiten hinausgeht.

Flexible Arbeitszeitmodelle und innovative Organisationsformen rücken daher zunehmend in den Fokus wissenschaftlicher und gesundheitspolitischer Debatten [5]. Sie sind wichtige Ansatzpunkte, um die Vereinbarkeit von Beruf und Privatleben zu verbessern, die Attraktivität der ärztlichen Tätigkeit zu steigern und die Berufszufriedenheit trotz hoher Arbeitsbelastung langfristig zu sichern [6–8]. Gleichzeitig verändern sich die Erwartungen an moderne Arbeitsplätze, insbesondere hinsichtlich flexibler Arbeitszeiten und besserer Vereinbarkeit.

Während das Zusammenspiel von Arbeitsbelastung, Arbeitszeitmodellen und Berufszufriedenheit im Pflegebereich bereits untersucht wurde, liegen für Ärztinnen und Ärzte, insbesondere in der Urologie, bislang nur begrenzte wissenschaftliche Erkenntnisse vor [2, 6, 9–11].

Ziel war es daher, eine Bestandsaufnahme der in der deutschen Urologie tätigen Ärzteschaft zu erstellen. Hierfür wurde eine Mitgliederbefragung der Deutschen Gesellschaft für Urologie e. V. (DGU), des Bundesverbandes der deutschen Urologie e. V. (BvDU) sowie der German Society of Residents in Urology (GeSRU) durchgeführt. In der vorliegenden Arbeit wurde der Fokus auf die Arbeitsbelastung, die Zufriedenheit mit dem Arbeitszeitmodell sowie die Berufszufriedenheit gelegt. Dabei sollten mögliche Einflussfaktoren identifiziert und Ansatzpunkte sichtbar gemacht werden, die zur langfristigen Sicherung einer qualitativ hochwertigen urologischen Versorgung beitragen können.

## Material und Methoden

Zwischen Mai und August 2024 erstellte eine Arbeitsgruppe aus 12 Urologinnen der AG-Ärztinnen und Wissenschaftlerinnen in der Urologie der Deutschen Gesellschaft für Urologie (DGU) einen Fragebogen, um die aktuelle berufliche Situation der in der Urologie in Deutschland tätigen Ärztinnen und Ärzte zu untersuchen. Der Fragebogen wurde an die Verteiler der DGU (Mailverteiler ca. 5300 Adressen, bei insgesamt rund 8000 Mitgliedern), der GeSRU (ca. 2000 Mitglieder) sowie des BvDU (ca. 2500 Adressen) verschickt. Er wurde am 18.10.2024 versendet und konnte bis zum 31.12.2024 anonym beantwortet werden. Die Umfrage wurde von 999 Teilnehmenden beantwortet. Da es sich um eine anonyme Befragung von ärztlichen Kolleginnen und Kollegen ohne Patientenbezug handelte, war kein Ethikvotum erforderlich.

### Fragebogen und Endpunkte

Der Fragebogen umfasste 18 Fragen, darunter Multiple-Choice-Fragen, Ja- und/oder Nein-Fragen und Skalenfragen. Erfragt wurden demografische Informationen (Geschlecht, Alter, Bundesland), die berufliche Situation (Titel, wissenschaftliche Tätigkeit, operative Tätigkeit, berufliche Position, Art des Arbeitsortes, Träger des Arbeitsortes, Spezialisierung), die berufliche Zufriedenheit, die Arbeitszeit (Wochenarbeitszeit, Arbeitszeit inklusive Überstunden/Diensten), die Zufriedenheit mit dem Arbeitszeitmodell, die Arbeitsbelastung, die Vergütungszufriedenheit sowie die familiäre Situation (Kinder im eigenen Haushalt, Elternzeit).

Für die vorliegenden Analysen wurden drei Hauptendpunkte dichotomisiert: (1) Arbeitsbelastung („hoch“ oder „sehr hoch“ vs. „angemessen/niedrig/sehr niedrig“), (2) Zufriedenheit mit dem Arbeitszeitmodell („möchte weniger arbeiten“ vs. „zufrieden/möchte mehr arbeiten“), (3) berufliche Zufriedenheit („unzufrieden“ oder „sehr unzufrieden“ vs. „zufrieden/neutral“). Altersgruppen wurden definiert als < 40, 40–55 und > 55 Jahre.

Das primäre Ziel der vorliegenden Auswertung war eine deskriptive Darstellung der aktuellen Situation der in der Urologie tätigen Ärztinnen und Ärzte in Deutschland. Des Weiteren wurden uni- und multivariable Analysen zu Korrelationen zwischen Arbeitsbelastung, Zufriedenheit mit dem Arbeitszeitmodell sowie beruflicher Zufriedenheit mit verschiedenen möglichen Einflussfaktoren durchgeführt.

### Statistische Analysen

Die deskriptive Statistik umfasste Häufigkeiten und Proportionen für kategoriale Variablen. Für kontinuierlich kodierte Variablen wurden Mittelwerte, Mediane und Spannweiten angegeben. Die statistische Signifikanz der Unterschiede in den Proportionen und Medianen wurde mit dem Wilcoxon-Rangsummentest, dem χ^2^-Test nach Pearson und dem Test nach Fisher bewertet.

Ein *p*-Wert von ≤ 0,05 wurde als statistisch signifikant angesehen. Es wurden univariable und multivariable logistische Regressionsanalysen durchgeführt, um Einflussfaktoren auf die Arbeitsbelastung, die Zufriedenheit mit dem Arbeitszeitmodell sowie die berufliche Zufriedenheit zu bewerten. Alle in der univariablen Analyse signifikanten Variablen (*p* ≤ 0,05) wurden in die multivariablen Modelle eingeschlossen. Alle Analysen wurden mit der Software R Version 4.5.1 (R-Projekt, R Foundation for Statistical Computing, Wien, Österreich) durchgeführt.

## Ergebnisse

Insgesamt beantworteten 999 Ärztinnen und Ärzte den Fragebogen (Tab. 1). Hiervon waren 66 % männlich, 34 % weiblich und eine Person (0,1 %) divers, das mediane Alter lag bei 47 Jahren. Die meisten Teilnehmer kamen aus Nordrhein-Westfalen (21 %), gefolgt von Bayern (17 %) und Baden-Württemberg (10 %). Einen Doktortitel hatten 61 %, 8 % waren habilitiert (4 % mit Professorentitel) und 19 % gingen aktuell einer wissenschaftlichen Tätigkeit nach. Die Mehrheit (73 %) war operativ tätig, 67 % gaben einen allgemeinurologischen und 36 % einen konservativ-onkologischen Schwerpunkt an. Überwiegend ambulant arbeiteten 53 %, stationär 43 % und in Wissenschaft, Privatwirtschaft, Industrie oder einer Behörde 2 % der Befragten. Während 74 % in Vollzeit (≥ 40 h/Woche) arbeiteten (82 % der Männer und 57 % der Frauen), gaben insgesamt 54 % an, inklusive Überstunden/Bereitschaftsdiensten ≥ 50 h/Woche zu arbeiten. Im Hinblick auf die familiäre Situation lebten 50 % mit ≥ 1 Kind unter 18 Jahren im selben Haushalt. Elternzeit hatten 31 % der Befragten bereits genommen, davon 56 % länger als 6 Monate (Tab. 1).

**Tab. 1:** Charakteristika der Gesamtkohorte (*n* = 999), Antworten der 18 Fragen.

Fragen des Fragebogens	*N* = 999
*1. Welches Geschlecht haben Sie? (n [%])*	
M	663 (66 %)
W	335 (34 %)
D	1 (0,1 %)
*2. Wie alt sind Sie? (in Jahren), Median (Interquartilsabstand)*	47 (37, 58)
*3. In welchem Bundesland arbeiten Sie oder haben Sie bis zuletzt gearbeitet? (n [%])*	
Baden-Württemberg	103 (10 %)
Bayern	170 (17 %)
Berlin	71 (7,1 %)
Brandenburg	24 (2,4 %)
Bremen	11 (1,1 %)
Hamburg	36 (3,6 %)
Hessen	79 (7,9 %)
Mecklenburg-Vorpommern	24 (2,4 %)
Niedersachsen	61 (6,1 %)
Nordrhein-Westfalen	205 (21 %)
Rheinland-Pfalz	48 (4,8 %)
Saarland	15 (1,5 %)
Sachsen	43 (4,3 %)
Sachsen-Anhalt	22 (2,2 %)
Schleswig-Holstein	38 (3,8 %)
Thüringen	35 (3,5 %)
Deutschsprachiges Ausland	14 (1,4 %)
*4. Was ist Ihr höchster akademischer Titel? (n [%])*	
Planmäßige Professur (durch Berufungsverfahren ausgewählt)	5 (0,5 %)
Außerplanmäßige Professur	39 (3,9 %)
Privatdozent/Privatdozentin	39 (3,9 %)
Dr. med	605 (61 %)
Dr. med. univ	16 (1,6 %)
Dipl. med	9 (0,9 %)
Sonstiger Titel	20 (2,0 %)
Kein Titel	266 (27 %)
*5. Gehen Sie aktuell einer wissenschaftlichen Tätigkeit nach? (n [%])*	
Ja	194 (19 %)
Nein	805 (81 %)
*6. Gehen Sie aktuell einer operativen Tätigkeit nach? (n [%])*	
Ja	727 (73 %)
Nein	272 (27 %)
*7. Welche Beschreibung der beruflichen Tätigkeit kommt Ihrer am nächsten? (n [%])*	
Niedergelassen mit operativer Tätigkeit	291 (29 %)
Niedergelassen ohne operative Tätigkeit	133 (13 %)
Angestellt in einer ambulanten Einrichtung (Praxis, MVZ, o. ä.) mit operativer Tätigkeit	52 (5,2 %)
Angestellt in einer ambulanten Einrichtung (Praxis, MVZ, o. ä.) ohne operative Tätigkeit	52 (5,2 %)
Angestellt in einer Klinik als Assistenzarzt/Assistenzärztin	164 (16 %)
Angestellt in einer Klinik als Facharzt/Fachärztin	77 (7,7 %)
Angestellt in einer Klinik als Oberarzt/Oberärztin	97 (9,7 %)
Angestellt in einer Klinik in Leitungsposition (stellvertr. Klinikdirektor/Klinikdirektorin, ltd. OA/OÄ, o. ä.)	42 (4,2 %)
Angestellt in einer Klinik als Chefarzt/Chefärztin	48 (4,8 %)
Behörde (z. B. Gesundheitsamt, MDK)	5 (0,5 %)
Privatwirtschaft/Industrie	6 (0,6 %)
Wissenschaft/Forschung/Lehre	5 (0,5 %)
Keine der oben genannten/andere/mehrere Tätigkeiten	27 (2,7 %)
*7.a In welcher Art von Einrichtung arbeiten Sie? (n [%])*	
Einzelpraxis	202 (20 %)
Gemeinschaftspraxis bzw. Praxisgemeinschaft	282 (28 %)
Medizinisches Versorgungszentrum (MVZ)	43 (4,3 %)
Grundversorger (Innere Medizin und/oder Chirurgie)	6 (0,6 %)
Regelversorger (Innere Medizin und Chirurgie + weitere Fachabteilung)	104 (10 %)
Schwerpunktversorger (obige + Kinderheilkunde und/oder Neurologie)	98 (9,8 %)
Maximalversorger (alle Fachabteilungen)	110 (11 %)
Universitätsklinikum (Maximalversorger + Lehre + Forschung)	112 (11 %)
Andere Einrichtung (Behörde, Privatwirtschaft, Wissenschaft, keine der genannten)	42 (4,2 %)
*7.b Zu welcher Gruppe zählt der Träger Ihrer Einrichtung? (n [%])*	
Freigemeinnütziger Träger (z. B. karitative oder kirchliche Häuser)	127 (13 %)
Öffentlicher Träger (z. B. kommunale Häuser, Universitätsklinika)	250 (25 %)
Privater Träger (z. B. Sana, Helios, Asklepios …)	88 (8,8 %)
Privatklinik	16 (1,6 %)
Selbstständiger Träger (z. B. eigene Praxis)	451 (45 %)
Sonstiger Träger	24 (2,4 %)
Andere Einrichtung (Behörde, Privatwirtschaft, Wissenschaft, keine der genannten)	43 (4,3 %)
*8. Wie zufrieden sind Sie mit Ihrer derzeitigen beruflichen Tätigkeit? (n [%])*	
Sehr zufrieden	151 (15 %)
Zufrieden	409 (41 %)
Weder zufrieden noch unzufrieden	200 (20 %)
Unzufrieden	191 (19 %)
Sehr unzufrieden	48 (4,8 %)
*9. Wie viele Wochenarbeitsstunden arbeiten Sie aktuell durchschnittlich (ohne Überstunden)? (n [%])*	
< 10 h/Woche (< 25 %)	22 (2,2 %)
10–19 h/Woche (Teilzeit; 25 bis ~45 %)	24 (2,4 %)
20–29 h/Woche (Teilzeit; 50 bis ~70 %)	75 (7,5 %)
30–39 h/Woche (Teilzeit; 75 bis ~95 %)	142 (14 %)
40 h/Woche oder mehr (Vollzeit)	736 (74 %)
*10. Wie viele Wochenarbeitsstunden arbeiten Sie aktuell tatsächlich im Durchschnitt (einschließlich Überstunden und Dienste)? (n [%])*	
< 10 h/Woche	30 (3,0 %)
10–19 h/Woche	22 (2,2 %)
20–29 h/Woche	47 (4,7 %)
30–39 h/Woche	113 (11 %)
40–49 h/Woche	243 (24 %)
50–59 h/Woche	314 (31 %)
≥ 60 h/Woche	230 (23 %)
*11. Sind Sie mit Ihrem Arbeitszeitmodell zufrieden? (n [%])*	
Ja	439 (44 %)
Nein – ich würde gerne mehr arbeiten	20 (2,0 %)
Nein – ich würde gerne weniger arbeiten	540 (54 %)
*12. Wie empfinden Sie Ihre aktuelle Arbeitsbelastung? (n [%])*	
Sehr hoch	344 (34 %)
Hoch	457 (46 %)
Angemessen	169 (17 %)
Niedrig	20 (2,0 %)
Sehr niedrig	9 (0,9 %)
*13. Wie zufrieden sind Sie mit Ihrer Vergütung? (n [%])*	
Sehr zufrieden	84 (8,4 %)
Zufrieden	394 (39 %)
Weder zufrieden noch unzufrieden	215 (22 %)
Unzufrieden	238 (24 %)
Sehr unzufrieden	68 (6,8 %)
*14. Wie viele Kinder (<* *18 Jahre) leben in Ihrem Haushalt? (n [%])*	
0	501 (50 %)
1	167 (17 %)
2	230 (23 %)
3	83 (8,3 %)
4	17 (1,7 %)
≥ 5	1 (0,1 %)
*15. Haben Sie bereits Elternzeit in Anspruch genommen? (n [%])*	
Nein	685 (69 %)
Ja – insgesamt nicht mehr als 2 Monate	70 (7,0 %)
Ja – insgesamt mehr als 2 Monate aber nicht mehr als 6 Monate	68 (6,8 %)
Ja – insgesamt mehr als 6 Monate aber nicht mehr als 1 Jahr	70 (7,0 %)
Ja – insgesamt mehr als 1 Jahr	57 (5,7 %)
Ja – insgesamt mehr als 2 Jahre aber nicht länger als 3 Jahre	36 (3,6 %)
Ja – insgesamt mehr als 3 Jahre	13 (1,3 %)
*16. In welchem Bereich der Urologie sind Sie spezialisiert oder hauptsächlich tätig? (n [%])*	
Allgemeine Urologie	671 (67 %)
Endourologie	132 (13 %)
Kinderurologie	31 (3,1 %)
Andrologie	66 (6,6 %)
Neurourologie	22 (2,2 %)
Konservative Uroonkologie	361 (36 %)
Operative Uroonkologie	163 (16 %)
Rekonstruktive Urologie	36 (3,6 %)
Andere	34 (3,4 %)

**Abb. 1:**
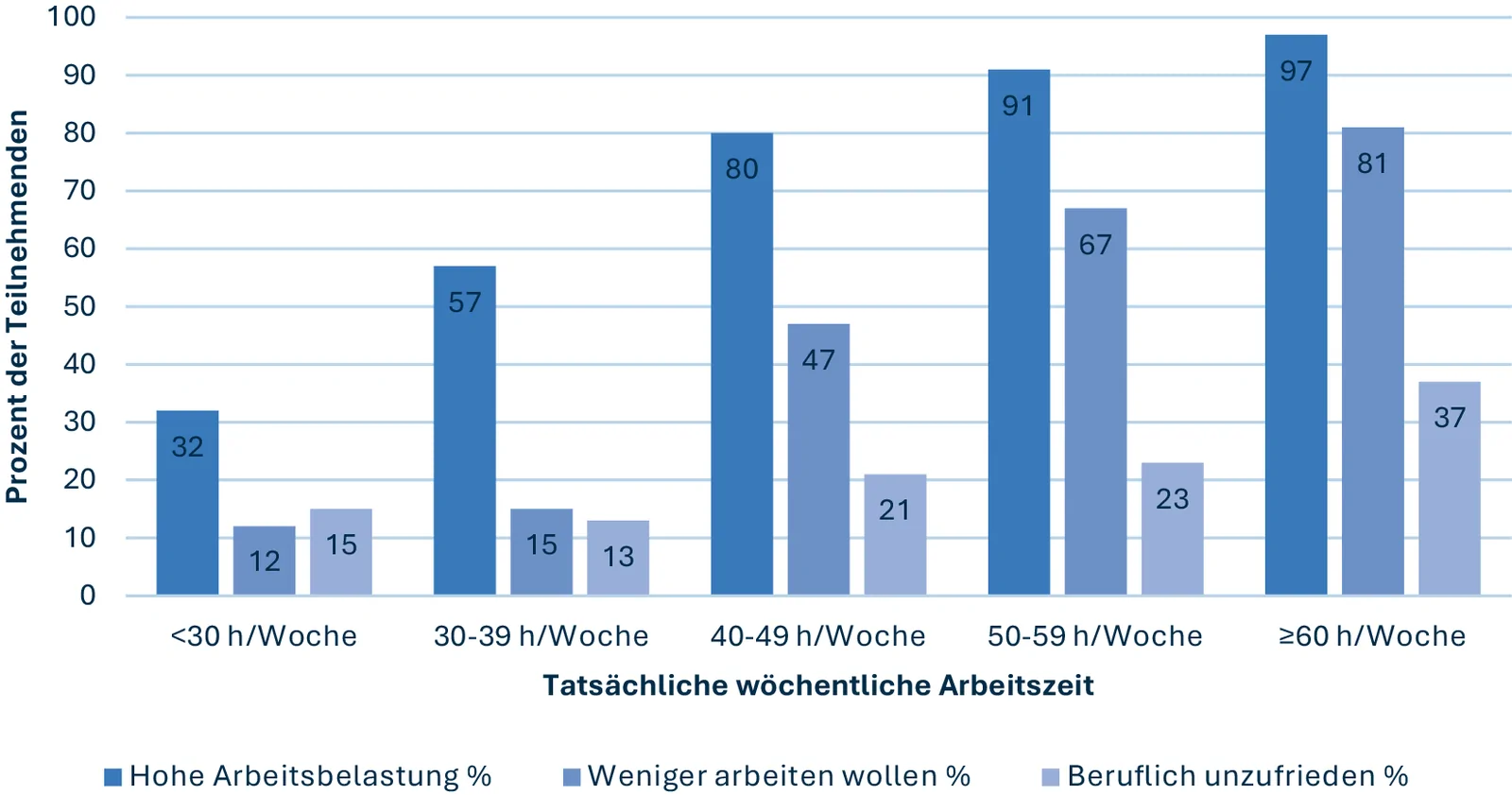
Belastung, Arbeitszeitwunsch und berufliche Zufriedenheit in Abhängigkeit der tatsächlichen Wochenarbeitszeit (inklusive Dienste und Überstunden)

Insgesamt beschrieben 80 % eine hohe oder sehr hohe Arbeitsbelastung und 54 % gaben an, ihre Arbeitszeit reduzieren zu wollen. Gleichzeitig waren 56 % zufrieden oder sehr zufrieden mit ihrer aktuellen beruflichen Situation. Die drei Endpunkte zeigten signifikante Korrelationen untereinander (r = 0,167–0,449; alle *p* < 0,001; Abb. 1).

### Uni- und multivariable logistische Regressionsmodelle

In univariablen logistischen Regressionsmodellen mit dem Endpunkt Arbeitsbelastung (hoch/sehr hoch) zeigten sich mehrere unabhängige Prädiktoren. Mit steigendem Alter nahm die Wahrscheinlichkeit hoher Arbeitsbelastung leicht ab (OR: 0,98). Nicht-operative Tätigkeit (OR: 0,42) und eine ambulant angestellte Position (OR: 0,34) wirkten protektiv. Sowohl die vertraglich vereinbarte Arbeitszeit (30–39 h OR: 3,75; ≥ 40 h OR: 10,46) als auch die tatsächliche Arbeitszeit waren starke Prädiktoren: Mit zunehmender Stundenzahl stieg das Risiko für hohe Arbeitsbelastung deutlich an – von OR 2,73 bei 30–39 h/Woche über OR 8,28 bei 40–49 h/Woche und OR 22,26 bei 50–59 h/Woche bis OR 78,17 bei ≥ 60 h/Woche. Auch die Vergütungszufriedenheit war eng mit der empfundenen Belastung assoziiert: Je unzufriedener die Befragten mit ihrer Vergütung, desto höher das Risiko für hohe Arbeitsbelastung (zufrieden OR: 1,76; weder noch OR: 2,53; unzufrieden OR: 7,1; sehr unzufrieden OR: 13,3). Weitere unabhängige Prädiktoren waren Kinder im Haushalt (OR: 1,38) sowie berufliche Unzufriedenheit (OR: 3,6). Der Wunsch nach Arbeitszeitreduzierung erhöhte das Risiko für hohe Arbeitsbelastung deutlich (OR: 17,8), während der Wunsch nach mehr Arbeitszeit protektiv wirkte (OR: 0,33).

In univariablen logistischen Regressionsmodellen mit dem Endpunkt Zufriedenheit mit dem Arbeitszeitmodell (weniger arbeiten wollen) zeigte sich ein ähnliches Muster. Mit steigendem Alter (OR: 0,98) und bei Männern (OR: 0,71) war der Wunsch nach Arbeitszeitreduzierung geringer ausgeprägt. Nicht-operative Tätigkeit wirkte ebenfalls protektiv (OR: 0,52). Höhere berufliche Positionen gingen durchgehend mit einem geringeren Wunsch nach Arbeitszeitreduzierung einher – am deutlichsten bei ambulant Angestellten (OR: 0,13), gefolgt von Chefärzten und Leitenden (OR: 0,33), niedergelassenen Ärztinnen und Ärzten (OR: 0,62) sowie Fachärzten (OR: 0,54). Tätigkeit an einem Grund- oder Regelversorger bzw. Schwerpunktversorger war im Vergleich zum Universitätsklinikum mit einem höheren Wunsch nach Arbeitszeitreduzierung assoziiert (OR jeweils 1,87 bzw. 1,88). Sowohl die vertraglich vereinbarte Arbeitszeit (30–39 h OR: 3,14; ≥ 40 h OR: 12,16) als auch die tatsächliche Arbeitszeit waren starke Prädiktoren (30–39 h OR: 1,28; 40–49 h OR: 6,51; 50–59 h OR: 14,43; ≥ 60 h OR: 31,52). Vergütungsunzufriedenheit (zufrieden OR: 1,73; weder noch OR: 3,68; unzufrieden OR: 6,94; sehr unzufrieden OR: 8,1), die Arbeitsbelastung (hoch OR: 0,22; angemessen OR: 0,02; niedrig OR: 0,01) sowie berufliche Unzufriedenheit (OR: 5,2) waren ebenfalls unabhängige Prädiktoren.

In univariablen logistischen Regressionsmodellen mit dem Endpunkt berufliche Zufriedenheit (unzufrieden/sehr unzufrieden) erwiesen sich wissenschaftliche Tätigkeit (OR: 2,11) und eine Arbeitszeit von ≥ 60 h/Woche (OR: 3,34) als Risikofaktoren, während Tätigkeit an einem Universitätsklinikum protektiv wirkte (OR: 0,57) und Frauen tendenziell zufriedener waren (OR: 0,71). Den stärksten Einfluss hatte die Vergütungszufriedenheit: Bei sehr unzufriedener Vergütung war das Risiko für berufliche Unzufriedenheit um das 36-Fache erhöht (weder noch OR: 3,9; unzufrieden OR: 8,8; sehr unzufrieden OR: 36,1). Auch der Wunsch nach Arbeitszeitveränderung in beide Richtungen (weniger arbeiten: OR 5,9; mehr arbeiten: OR 5,7) sowie die Arbeitsbelastung (hoch OR: 0,24; angemessen OR: 0,11) waren unabhängige Prädiktoren.

In multivariablen logistischen Regressionsanalysen blieben nach Adjustierung für alle univariabel signifikanten Variablen verschiedene unabhängige Prädiktoren bestehen (Tab. 2).

**Tab. 2:** Multivariable Analysen verschiedener Variablen mit den drei Endpunkten Arbeitsbelastung, Zufriedenheit mit dem Arbeitszeitmodell sowie beruflicher Zufriedenheit

Einflussfaktor	Arbeitsbelastung (hohe Belastung) OR (95 %-KI), *p*-Wert	Zufriedenheit mit dem Arbeitszeitmodell (unzufrieden) OR (95 %-KI), *p*-Wert	Berufliche Zufriedenheit (unzufrieden) OR (95 %-KI), *p*-Wert
Alter (pro Jahr)	1,00 (0,98–1,03), 0,726	1,00 (0,98–1,02), 0,991	–
*Geschlecht (Ref: Männlich)*			
Weiblich	–	1,02 (0,68–1,55), 0,907	1,01 (0,67–1,50), 0,979
*Operative Tätigkeit (Ref: Ja)*			
Nein	1,32 (0,79–2,24), 0,290	0,99 (0,62–1,58), 0,958	–
Wissenschaftliche Tätigkeit (Ref: Ja):	–	–	–
Nein	–	–	2,32 (1,29–4,32), 0,006
*Position (Ref: Assistenzarzt)*			
Facharzt	2,58 (0,98–7,09), 0,059	0,59 (0,28–1,24), 0,163	–
Oberarzt	3,32 (1,20–9,76), 0,024	0,72 (0,36–1,48), 0,374	–
Chefarzt/Leitung	2,12 (0,75–6,25), 0,164	0,24 (0,10–0,54), 0,001	–
Niedergelassen	0,11 (0,00–12,6), 0,592	2,16 (0,03–152), 0,788	–
Ambulant angestellt	0,13 (0,00–15,1), 0,622	1,43 (0,02–100), 0,901	–
Sonstige	22573 (0,00–NA), 0,985	0,00 (0,00–0,00), 0,992	–
*Versorgungsebene (Ref: Ambulant/Praxis)*			
Grund‑/Regelversorger	0,05 (0,00–5,72), 0,475	6,52 (0,08–441), 0,511	1,04 (0,57–1,85), 0,906
Maximalversorger	0,03 (0,00–2,67), 0,376	2,53 (0,03–162), 0,744	1,02 (0,53–1,89), 0,956
Schwerpunktversorger	0,03 (0,00–3,65), 0,407	4,11 (0,05–277), 0,620	2,62 (1,45–4,72), 0,001
Universitätsklinikum	0,03 (0,00–3,03), 0,394	2,30 (0,03–147), 0,770	1,13 (0,53–2,39), 0,743
Sonstige (Behörde, Privatwirtschaft, etc.)	0,00 (NA‑1,66e + 41), 0,979	1674105 (0,00–NA), 0,992	0,71 (0,14–2,79), 0,649
*Tatsächliche Arbeitszeit (Ref: <* *30 h)*			
30–39 h	3,00 (1,51–6,06), 0,002	1,04 (0,38–2,89), 0,944	1,14 (0,43–3,06), 0,792
40–49 h	6,73 (3,43–13,5), < 0,001	4,25 (1,82–10,5), 0,001	1,15 (0,49–2,84), 0,756
50–59 h	20,9 (9,22–49,2), < 0,001	8,06 (3,38–20,3), < 0,001	0,90 (0,37–2,28), 0,813
≥ 60 h	66,1 (22,5–222), < 0,001	15,6 (6,13–41,6), < 0,001	1,26 (0,51–3,28), 0,628
*Vergütungszufriedenheit (Ref: sehr zufrieden)*			
Zufrieden	1,20 (0,61–2,33), 0,589	1,02 (0,50–2,12), 0,958	1,12 (0,47–3,14), 0,809
Weder noch	0,98 (0,46–2,08), 0,960	1,37 (0,63–2,99), 0,433	2,81 (1,18–7,85), 0,029
Unzufrieden	2,96 (1,22–7,34), 0,017	1,66 (0,75–3,69), 0,215	5,45 (2,33–15,0), < 0,001
Sehr unzufrieden	7,21 (1,60–45,3), 0,018	1,00 (0,36–2,89), 0,995	24,5 (9,05–76,4), < 0,001
Keine Kinder im Haushalt	1,57 (0,99–2,52), 0,057	–	–
Andere Endpunkte:			
*Arbeitsbelastung (Ref: sehr hoch)*			
Hoch	–	0,39 (0,26–0,59), < 0,001	0,36 (0,24–0,53), < 0,001
Angemessen	–	0,09 (0,04–0,17), < 0,001	0,42 (0,20–0,87), 0,023
Niedrig	–	0,05 (0,00–0,41), 0,016	0,78 (0,15–3,54), 0,756
Sehr niedrig	–	0,00 (NA‑6,11e + 11), 0,974	0,85 (0,05–8,72), 0,897
*Zufriedenheit mit Arbeitszeitmodell (Ref: Ja)*			
Weniger arbeiten wollen	6,10 (3,43–11,4), < 0,001	–	2,84 (1,75–4,70), < 0,001
Mehr arbeiten wollen	0,58 (0,18–1,75), 0,339	–	4,48 (1,30–14,3), 0,013
*Berufliche Zufriedenheit (Ref: sehr zufrieden)*			
Unzufrieden	2,04 (1,22–3,45), 0,007	3,44 (1,91–6,41), < 0,001	–
Weder noch	3,29 (1,50–7,48), 0,004	9,72 (4,91–19,9), < 0,001	–
Unzufrieden	2,13 (0,93–5,03), 0,079	11,2 (5,38–24,0), < 0,001	–
Sehr unzufrieden	3,62 (0,63–36,4), 0,203	26,4 (7,28–126), < 0,001	–

*OR* Odds Ratio, *KI* Konfidenzintervall

Arbeitszeit war der stärkste Prädiktor für hohe Arbeitsbelastung, mit einer OR von 66,08 bei ≥ 60 h/Woche. Vergütungsunzufriedenheit blieb ebenfalls signifikant (unzufrieden OR: 2,96; sehr unzufrieden OR: 7,2). Unter den beruflichen Positionen wiesen Oberärzte gegenüber Assistenzärzten ein mehr als 3‑fach erhöhtes Risiko für hohe Arbeitsbelastung auf (OR: 3,32). Der Wunsch nach Arbeitszeitreduzierung war stark assoziiert (OR: 6,10), während der Wunsch nach mehr Arbeitszeit protektiv wirkte (OR: 0,58).

Für den Endpunkt Wunsch nach Arbeitszeitreduzierung blieb die tatsächliche Arbeitszeit der dominante Faktor (≥ 60 h OR: 15,58). Leitende Positionen waren mit einem geringeren Wunsch nach Reduktion assoziiert (OR: 0,24). Berufliche Unzufriedenheit zeigte eine stark dosisabhängige Assoziation (bis OR: 26,40), während die Arbeitsbelastung protektiv wirkte.

Für die berufliche Unzufriedenheit war die Vergütungszufriedenheit der stärkste Prädiktor – bei sehr unzufriedener Vergütung war das Risiko für berufliche Unzufriedenheit um das 24-Fache erhöht. Wissenschaftliche Tätigkeit (OR: 2,32) und Tätigkeit an einem Schwerpunktversorger (OR: 2,61) blieben ebenfalls unabhängige Risikofaktoren. Der Wunsch nach Arbeitszeitveränderung in beide Richtungen war assoziiert (weniger arbeiten OR: 2,84; mehr arbeiten OR: 4,48). Hohe Arbeitsbelastung war mit einem geringeren Risiko für berufliche Unzufriedenheit assoziiert.

## Diskussion

Die Ergebnisse der vorliegenden Befragung liefern Daten zur beruflichen Situation von Urologinnen und Urologen in Deutschland. Laut Ärztestatistik der Bundesärztekammer zum 31.12.2024 gibt es in Deutschland 6762 berufstätige Fachärztinnen und Fachärzte für Urologie (9000 Registrierte insgesamt), mit einem Frauenanteil von 23,2 % unter den Berufstätigen [14]. Hinzu kommen schätzungsweise noch etwa 2500–3000 Ärztinnen und Ärzte in urologischer Weiterbildung.

Mit einer großen Stichprobe von knapp 1000 Teilnehmern aus allen Versorgungsebenen bietet unsere Studie daher eine breite Datengrundlage für die Bewertung der aktuellen Arbeitssituation in der deutschen Urologie.

### Hohe Arbeitsbelastung als zentraler Aspekt

Mit 80 % gibt eine große Mehrheit der Befragten an, eine hohe oder sehr hohe Arbeitsbelastung zu empfinden, was die beschriebenen strukturellen Probleme des deutschen Gesundheitssystems unterstreicht [12]. Diese Belastungsrate liegt deutlich über vergleichbaren Erhebungen in anderen medizinischen Fachrichtungen und bestätigt die besonderen Herausforderungen operativer Fächer [8]. Besonders bemerkenswert ist der starke Zusammenhang zwischen tatsächlicher Arbeitszeit und empfundener Belastung: Während Ärztinnen und Ärzte mit weniger als 30 Wochenstunden Arbeitszeit in 32 % eine hohe oder sehr hohe Arbeitsbelastung angaben, steigt dieser Anteil bei über 60 Wochenstunden auf etwa das 3‑Fache an – das entspricht 97 % der Ärztinnen und Ärzte in dieser Gruppe.

Die Identifikation der beruflichen Position als unabhängiger Prädiktor verdeutlicht die hierarchiespezifischen Belastungsunterschiede. Oberärzte zeigten ein mehr als dreifach erhöhtes Risiko für eine hohe Arbeitsbelastung im Vergleich zu Assistenzärzten. Dies könnte die besondere Position dieser Berufsgruppe zwischen Weiterbildungsverantwortung, klinischen Aufgaben und administrativen Tätigkeiten widerspiegeln.

### Diskrepanz zwischen Arbeitszeit und Zufriedenheit

Ein interessanter Befund der Studie ist die Tatsache, dass trotz hoher Arbeitsbelastung (80 %) und dem weit verbreiteten Wunsch nach Arbeitszeitreduzierung (54 %) noch 56 % der Urologen mit ihrem Beruf zufrieden oder sehr zufrieden sind.

Diese scheinbare Diskrepanz könnte mehrere Ursachen haben: Zum einen spiegelt sie das traditionelle Verständnis des ärztlichen Berufs als Berufung wider, bei dem hohe Arbeitsbelastung als inhärenter Bestandteil der Tätigkeit akzeptiert wird [13]. Zum anderen verdeutlicht sie möglicherweise die intrinsische Motivation und Sinnhaftigkeit der urologischen Tätigkeit, die trotz struktureller Probleme zur Berufszufriedenheit beiträgt. Der dennoch vorhandene Wunsch von 54 % der Befragten nach Arbeitszeitreduzierung verdeutlicht jedoch den Handlungsbedarf bei der Gestaltung nachhaltiger und flexibler Arbeitszeitmodelle. Die multivariable Analyse zeigt, dass insbesondere leitende Ärzte und Chefärzte (OR: 0,24) ein geringeres Risiko für den Wunsch nach Arbeitszeitreduzierung aufweisen, was auf die größeren Gestaltungsmöglichkeiten in Führungspositionen hindeuten könnte.

### Geschlechtsspezifische Unterschiede und Vereinbarkeit

Die Ergebnisse bestätigen zudem bekannte geschlechtsspezifische Unterschiede in der Arbeitswelt: Während 82 % der Männer in Vollzeit arbeiten, gilt dies nur für 57 % der Frauen. Diese Differenz reflektiert vermutlich unterschiedliche Strategien zur Vereinbarkeit von Beruf und Familie zwischen den Geschlechtern, zumal 50 % der Befragten mit mindestens einem Kind unter 18 Jahren im selben Haushalt lebten. Die Tatsache, dass 31 % bereits Elternzeit genommen haben – davon 56 % länger als 6 Monate –, zeigt eine zunehmende Sensibilität für Work-Life-Balance-Aspekte in der Urologie [7].

In unserer Befragung lag der Frauenanteil bei 34 % und damit über dem in der offiziellen Ärztestatistik ausgewiesenen Anteil von 23,2 % [14]. Dieser Befund lässt sich als Abbild aktueller Entwicklungen im Fach interpretieren. Eine Untersuchung zeigt, dass Ärztinnen bis zur Facharztprüfung nahezu paritätisch vertreten sind, in Führungspositionen und der universitären Forschung jedoch weiterhin deutlich unterrepräsentiert bleiben [15]. Damit verdeutlicht unsere Studie nicht nur die aktuelle Arbeitsbelastung und Arbeitszeitmodelle, sondern auch die dynamische geschlechtsspezifische Entwicklung innerhalb der Urologie.

### Vergütung als Zufriedenheitsfaktor

Die starke Assoziation zwischen der Vergütungszufriedenheit und den untersuchten Endpunkten unterstreicht die Bedeutung angemessener Entlohnung für die Berufszufriedenheit. Auffällig ist die Odds Ratio von 24,52, die sehr unzufriedene Vergütung mit beruflicher Unzufriedenheit assoziiert. Dies verdeutlicht, dass finanzielle Wertschätzung ein wichtiger Faktor für die langfristige Bindung qualifizierter Urologen an das Fach darstellt.

### Limitationen

Die vorliegende Studie weist mehrere Limitationen auf. Als Querschnittstudie können keine kausalen Zusammenhänge etabliert werden, dennoch erlaubt die große und heterogene Stichprobe eine aussagekräftige Bestandsaufnahme. Die Einschätzungen zur Arbeitsbelastung und Zufriedenheit beruhen auf Selbstauskünften, die mögliche Verzerrungen einschließen, gleichzeitig aber authentische Einblicke in die subjektiv wahrgenommene Arbeitssituation geben. Zudem könnte ein Selektionsbias vorliegen, da möglicherweise überdurchschnittlich belastete oder unzufriedene Ärzte eher an der Umfrage teilgenommen haben. Weiterhin kann die Dichotomisierung der kontinuierlichen Endpunkte zu Informationsverlust führen, was ebenso berücksichtigt werden sollte. Eine exakte Rücklaufquote konnte aufgrund der Nutzung mehrerer Verteiler nicht bestimmt werden, doch mit 999 Teilnehmenden liegt die Zahl deutlich über vergleichbaren Erhebungen in ärztlichen Fachgruppen. Der im Vergleich zur offiziellen Ärztestatistik höhere Anteil weiblicher Teilnehmender dürfte die zunehmende Zahl von Ärztinnen in Weiterbildung widerspiegeln, die dort nicht gesondert erfasst sind, und macht die Ergebnisse besonders aktuell und relevant [15].

### Schlussfolgerung

Die Studie gibt eine Übersicht über die Zusammenhänge zwischen Arbeitsbelastung, Zufriedenheit mit dem Arbeitszeitmodell und Berufszufriedenheit in der deutschen Urologie. Der mehrheitliche Wunsch nach Reduktion der tatsächlichen Arbeitszeit zeigt jedoch den Handlungsbedarf für strukturelle Anpassungen auf, damit die Attraktivität der Urologie langfristig gesichert werden kann und eine qualitativ hochwertige Patientenversorgung gewährleistet bleibt. Die gewonnenen Erkenntnisse sollten als Grundlage für gezielte Interventionen und weitere longitudinale Studien zur Weiterentwicklung der beruflichen Situation von Urologinnen und Urologen dienen.

## Fazit für die Praxis

- Hohe Arbeitsbelastung und der Wunsch nach Arbeitszeitreduktion sind in der deutschen Urologie weit verbreitet – strukturelle Anpassungen sind notwendig, um die Attraktivität des Fachs langfristig zu sichern.
- Gleichzeitig ist die Mehrheit der Befragten mit ihrem Beruf zufrieden; intrinsische Motivation und Identifikation mit der Urologie bleiben eine wichtige Ressource.
- Vergütungsunzufriedenheit ist der stärkste unabhängige Prädiktor für berufliche Unzufriedenheit – faire Entlohnung ist eine zentrale Voraussetzung für Nachwuchsgewinnung und -bindung.
- Leitende Positionen und der ambulante Sektor sind mit geringerer Belastungswahrnehmung assoziiert; flexible Karrierewege und Praxismodelle sollten als attraktive Option sichtbarer gemacht werden.
- Die Ergebnisse liefern eine aktuelle Bestandsaufnahme und sollten Ausgangspunkt für gezielte berufspolitische Maßnahmen und weiterführende longitudinale Untersuchungen sein.

## Data Availability

Die Daten sind auf begründete Anfrage bei dem korrespondierenden Autor erhältlich.
